# *Lysinibacillus macroides* stimulates systemic resistance against *Potyvirus papayanuli* in cucumber

**DOI:** 10.3389/fpls.2026.1780010

**Published:** 2026-03-25

**Authors:** Mohsen M. Elsharkawy, Faisal Ay Alzahrani

**Affiliations:** 1 Department of Agricultural Botany, Faculty of Agriculture, Kafrelsheikh University, Kafr Elsheikh, Egypt; 2 Department of Chemistry, College of Sciences and Arts, King Abdulaziz University, Rabigh, Saudi Arabia

**Keywords:** disease severity, ELISA, *lysinibacillus macroides*, Potyvirus papayanuli, real time PCR, rhizosphere soil

## Abstract

**Introduction:**

New and potentially useful rhizobacterial isolates are the focus of the most ongoing research into phytoviruses management because of their ability to inhibit viral replication.

**Methods:**

We investigated the efficacy of a newly-isolated strain of *Lysinibacillus macroides* in controlling *Potyvirus papayanuli* (papaya ring spot virus, PRSV) in cucumber plants.

**Results and discussion:**

Significant changes were observed between the treatment and control groups. For instance, cucumber plants were shown to have less severe viral symptoms after being pre-treated with *L. macroides* and then inoculated with PRSV. Upon application of *L. macroides* soil drenching (SD) treatment, the disease severity rate decreased significantly by 78.3% and 71.5% at 2- and 3- weeks post-infection (WPI), respectively. Plants treated with *L. macroides* had a lower PRSV titer using ELISA. Moreover, in PRSV-infected cucumbers, *L. macroides* treatments significantly increased antioxidant enzymes production and defense gene expressions as compared to the control treatment. Therefore, it is possible that *L. macroides* induces systemic resistance in cucumber plants and adversely impacts PRSV development. As a sustainable and eco-friendly approach for PRSV management, our study is the first report on the antiphytoviral activity of *L. macroides* against PRSV.

## 1 Introduction

Plant viruses are a constant threat to economically important crops around the world, but managing approaches are complicated (Anikina et al., 2023). Papaya production is in danger because of papaya viruses. *Potyvirus papayanuli* (papaya ring spot virus, PRSV) causes papaya ringspot disease, which is one of the most prevalent diseases that affect papaya trees (Maina et al., 2017). Infected papaya fields with PRSV can lose 40–100% of their crop yield (Thirugnanavel et al., 2015). Species belonging to the Cucurbitaceae family (including cucumber) are susceptible to systemic infection caused by PRSV (Rezende and Martins, 2005). The origin of the viral isolate and the species/varieties investigated determine cucurbit sensitivity to PRSV infection. Cucurbit species/varieties’ susceptibility to PRSV infection was confirmed by Mansilla et al. (2012). Additionally, the severity of PRSV on cucumber plants was illustrated (Elsharkawy and Mousa, 2015; Khalaf et al., 2019). The most common vector for PRSV transmission under the field conditions is aphids (Hidayat et al., 2022). Standard pesticides may not be effective against all aphids. The use of chemical pesticides also carries the risk of disrupting both humans and the environment (Fan et al., 2022). Geographic strain variations have impeded the development of transgenic plants exhibiting resistance to PRSV (Ruanjan et al., 2007; Akhtar et al., 2023). Therefore, PRSV management needs a cutting-edge strategy that is eco-friendly and reliable.

The detrimental effects of synthetic chemicals on humans and the environment attract researchers to biopesticides made from beneficial bacteria, which have the fewest side effects and may provide safe alternatives for the control of plant pathogens and viral infections. PGPR (plant growth-promoting rhizobacteria) are beneficial microorganisms that colonize plant roots, effectively modulating the plant’s defense mechanisms against diseases while also enhancing plant growth (Elsharkawy et al., 2022). They also induce a wide spectrum of immune responses against various pathogens, including bacteria, fungi, and viruses (Kumar et al., 2005). PGPR strains have been successfully employed in numerous studies to control plant diseases (Elsharkawy et al., 2022, 2023). Rhizosphere soil microorganisms can potentially affect plant development, and plants can subsequently modulate the microbial communities within this region (Van der Putten et al., 2013; Berendsen et al., 2018). Most research concerning the interactions between soil microorganisms and plant diseases has primarily concentrated on soil-borne pathogens (Yin et al., 2021; Zhou et al., 2022). Rhizosphere microorganisms are more prevalent in soil with healthy plants than in soil with sick plants (Huang et al., 2019). A strain of *Bacillus subtilis* was used to promote cucumber mosaic virus resistance in cucumber (Elsharkawy et al., 2022). The implementation of PGPR-mediated resistance demonstrated significant potential as a practical strategy for virus disease management in sustainable agriculture. Plant growth promotion may be triggered by *Lysinibacillus macroides*. The isolate of *L. macroides* significantly improved the growth and yield of *Solanum lycopersicum* L (Jyolsna et al., 2021). *Lysinibacillus* has been shown to have metal tolerance and the potential to stimulate plant development in polluted environments (Franchi et al., 2017). The antibacterial action against the pathogen *Xanthomonas campestris* was shown whenever *L. macrolides* were added either alone or as part of a consortium culture (Liu et al., 2016).

The objectives of our study were to evaluate the antiviral potential of *L. macroides* isolated from cucumber rhizosphere in protecting plants against PRSV infection, and to determine effective treatments for reducing the severity of PRSV disease. Indicators of oxidative stress, comprising enzymes responsible for neutralizing reactive oxygen species such as peroxidase, superoxide dismutase, and polyphenol oxidase, alongside the expression of defense-related genes, were employed to assess the resistance mechanisms in cucumber.

## 2 Material and methods

### 2.1 Isolation and identification of rhizobacteria

Twenty-three soil samples (from cucumber fields in Kafr Elsheikh Governorate, Egypt) were collected. Each sample was thoroughly vortexed prior to inoculation on nutrient agar. Plates were incubated at 30 °C for 48 h and subsequently stored at 4 °C. Each colony was properly isolated and placed onto new nutrient agar plates to eradicate any possible background flora and guarantee optimum isolation. The streaking approach was repeated five times or more. Forty purified distinct bacterial strains were grown on nutrient agar slants. The most efficient strain against PRSV was chosen from all the isolated strains. Physiological and morphological characteristics from Bergey’s Manuals were used together with universal primers for 16S ribosomal DNA to identify isolates (Bergey and Holt, 2000; Elsharkawy et al., 2022). The BigDye Ready Reaction kit was used to precisely prepare the samples for dye terminator cycle sequencing (Applied Biosystems, CA, USA). The sequences were uploaded to GenBank’s DNA sequences database and analyzed utilizing nucleotide alignment blast. Phylogenetic trees were constructed from sequence alignments utilizing MEGA 12 software (12^th^ version) and neighbor-joining algorithms (Tamura et al., 2011).

### 2.2 *In vivo* trial

Cucumber leaves displaying symptoms consistent with PRSV, such as pronounced mosaic patterns and foliar deformation, were gathered and subsequently utilized as fresh virus inoculum following confirmation of PRSV positivity using ELISA (Elsharkawy and Mousa, 2015). The investigation was conducted in a controlled environment free of insects within a net-house. The efficacy of three treatments of *L. macroides* in reducing the severity of PRSV was evaluated. The treatments consisted of the following: H (healthy), SP (seed biopriming), RD (root dipping), SD (soil drenching), and infected control. Each treatment was performed independently. The pot experiment was established using a totally randomized design, comprising three experimental replications, each involving 10 plants. The cucumber variety employed in this investigation was Beta alpha. All of the plants were inoculated with PRSV, except for the healthy group. *L. macroides* was inoculated into nutrient broth and incubated for a duration of 2 days. After 5 minutes of centrifugation of the *L. macroides* cultures at 6000 g, the supernatant was discarded. Once the *L. macroides* pellets were mixed again with SW (sterile water), a solution was made with a final concentration of 5 × 10^9^ CFU/mL.

Phosphoric acid (10%) was applied to the cucumber seeds after a complete wash in SW to remove any remaining fungicides. The seeds were cleaned again with SW and then allowed to dry naturally at room temperature overnight. For SP treatment, after an hour soaking of 50 cucumber seeds in 100 mL of *L. macroides* solution (containing 5 × 10^9^ CFU/mL), the seeds of cucumber were planted in plastic pots that contained 200 grams of peat moss. After growing cucumber seedlings for one week, they were taken out of the soil and thoroughly washed with clean water to remove any soil particles for the RD treatment. Following the previously stated process, the seedlings were submerged in an *L. macroides* suspension at a concentration of 5 × 10^9^ CFU/mL for 1 h before being planted in plastic pots (6-inch) with soilless potting mix. Seedlings that were just submerged in water served as a control group. Cucumber seedlings were planted into pots for the purpose of the SD treatment. Each pot received irrigation weekly with 25 mL of *L. macroides* suspension, which was poured around the bottom of the plant’s stem and saturated the soil. The course of treatment consisted of three applications separated one week apart. The pots in the untreated control plants were received only water. Mechanical inoculation of cucumber seedlings with PRSV was carried out using dusted carborundum (as an abrasive material) and K2HPO4 buffer (0.1 M), as described by Khalaf et al. (2019). The experimental plants were mechanically inoculated with PRSV one week after the *L. macroides* treatments.

### 2.3 Disease severity evaluation

The plants were kept in a temperature range of 25 ± 2°C and a relative humidity of 70-80% in the greenhouse. Following each treatment application, observations were taken on the disease’s severity in percentage form, beginning on the day symptoms first manifested. The following disease rating scale (explained by Elsharkawy and Mousa (2015) with some modifications) was used to assess viral symptoms at 2- and 3- weeks post virus inoculation (WPI) in both the treated and untreated control groups: No symptoms is indicated by 0, yellowing of the lower leaves (1), mosaic and yellowing (2), severe mosaic and mottling (3), and deformed leaves, stunted plant development, severe mosaic, or death of the plant (4).

The formula explained by Elsharkawy and Alzahrani (2026) was used to assess the severity of PRSV:


$$\text{DS}=\frac{\sum (Disease\;scale\;\times \;Number\;of\;plants\;per\;scale)}{(Total\;number\;of\;plants\;\times \;The\;highest\;disease\;scale)\;}\;\times \;100$$

As explained by Elsharkawy and Mousa (2015), ELISA was applied on the apical leaves of each treatment to ascertain if each treatment had an impact on the virus concentration. Two weeks after the final treatment, the test plants’ chlorophyll content was measured using the SPAD meter.

### 2.4 Assessment of the total soluble carbohydrates, proteins and ascorbic acid content

The total soluble carbohydrate (TSC) in groups of cucumber plants at two WPI was measured using the anthrone method, as described by Islam et al. (2020). The reaction was evaluated using absorbance at 625 nm, and a standard glucose curve was employed to calculate the TSC (mg/g dry weight). Additionally, each group’s concentration of total soluble protein was determined (Song et al., 2015; Abdelkhalek et al., 2022).

According to Abdelkhalek et al. (2022), the accumulated amount of AsA (ascorbic acid) in the cucumber groups was evaluated using Na-molybdate. The reaction’s absorbance was measured at 660 nm after 40 min. of incubation at 60 °C.

### 2.5 Free radicals and antioxidant enzymes evaluations

Following the protocol of Shimada et al. (1992), the following procedure was done to evaluate the free radical levels in the cucumber groups at 2-WPI using the PRSV challenge: A solution of 100 µL of leaves extract and 2 mL of 2,2-Diphenyl-1-picrylhydrazyl (0.05 M in methanol) was prepared. The scavenging activity (%) was determined by observing the decline in color at 517 nm for 30 min. This was accomplished by using the following formula:

Free radical scavenging (%) = (AI - A30/AI) × 100, where AI is the initial reaction absorbance and A30 is the reaction absorbance after 30 min.

To understand more about the antioxidant capacity of cucumber plants after being challenged with PRSV, leaf samples were collected from all four groups and evaluated them for three distinct enzymes at 2-WPI: peroxidase (POX), polyphenol oxidase (PPO), and superoxide dismutase (SOD). After drying, the leaves were homogenized using a phosphate buffer (0.1 M, pH 7.0), 100 mM Na-EDTA, and 1% w/v polyvinylpyrrolidone, resulting in a solid-to-liquid ratio of 1:4. Centrifugation at 5000 rpm for 10 min was used to extract different enzymes from the clear supernatant. Our approach to measuring SOD activity was based on a photocatalytic reduction experiment using nitro blue tetrazolium (NBT) chloride, as described by Beauchamp and Fridovich (1971) with a few modifications. Mixtures containing NBT (75 µM), EDTA (0.05 mM), methionine (13 mM), and riboflavin (20 µM) were added to with 100 µL of plant extract. The final volume was adjusted using 1.5 mL of potassium phosphate buffer (pH 7.8). To start the photochemical reaction, the mixture was placed under two 15- W fluorescent lights for 15 min at 25°C. Employing a 50% reduction in NBT color as an indicator of one unit of SOD activity, the color decline in the combination was assessed at 560 nm. Additionally, the quinone method was employed to evaluate PPO activity, consistent with the procedures of AHN and CHO (Cho and Ahn, 1999). After adding 0.5 mL of plant extract to 1 mL quinone (50 mM, pH of 6.0), which was made in Tris-HCl (100 mM), the mixture was incubated for 10 min. at 25 °C. Under these reaction conditions, a rise in absorbance of 0.001 at 420 nm was used to represent one unit of enzyme activity, which is measured in µM/g of fresh weight. Finally, the POX activity was evaluated using guaiacol and hydrogen peroxide, as described in Angelini et al. (1990). The 1.2 mL test reaction included the following components: 80 µL of plant extract, 120 µL of hydrogen peroxide (1 mM) and 0.5 mL of guaiacol (5 mM). The final volume was adjusted using a phosphate buffer (pH 7.0, 100 mM) before 10 min of incubation at 30 °C. The final color was measured at 480 nm.

### 2.6 The influence of *L. macroides* treatments on gene expression of cucumber plants

The procedures described by Elsharkawy et al. (2022) were followed to extract RNA from cucumber leaves at 2 days after PRSV inoculation. The reverse transcription (RT)-qPCR was performed using the primers (*PR-1, PR-3, PR5, LOX-1*, and *Actin*) provided in **Table 1**. Transcript levels relative to the housekeeping gene (*Actin*) of cucumber were normalized. A real-time PCR assay with SYBR Green was carried out in technical triplicate using a 7500 real-time PCR system (Applied Biosystems). The comparative 2^−ΔΔCt^ method technique was used to determine relative expression levels, using threshold values obtained from the ABI PRISM-7500 Software Tool.

**Table 1:** The RT-qPCR primer sequences.

Primer	Forward	Reverse
*PR-1*	TGCTCAACAATATGCGAACC	TCATCCACCCACAACTGAAC
*PR-3*	CACTGCAACCCTGACAACAACG	AAGTGGCCTGGAATCCGACTG
*PR-5*	CATTCTGCCTTTGTGCTTTTTC	ATTGATCGTCACGGTCTCGCC
*LOX-1*	CTCTTGGGTGGTGGTGTTTC	TGGTGGGATTGAAGTTAGCC
*Actin*	TGCTGGTCGTGACCTTACTG	GAATCTCTCAGCTCCGATGG

### 2.7 Effect of *L. macroides* on growth and yield of cucumber plants

A plastic house at the experimental farm of Kafrelsheikh University served as the setting for the two trials. After 45 days of transplanting, the fresh weight method was used to calculate the sixth leaf’s area (dm^2^). For this estimation, five discs with a surface area of 1.13 cm^2^ were extracted from the leaves and the formula (explained by Khalaf et al., 2019) was used:


$$\text{Leaf area}=\frac{\text{leaves fresh weight}\times \text{discs area}}{\text{Discs fresh weight }}\;$$

The fruit yield was determined by weighing the fruits in kg and the number of fruits per plant. The total quantity of fruits produced throughout both harvest seasons was determined (2024/25 and 2025/26 seasons). Based on the yield from both seasons, the number of pickings per plant and the fruit production in kg were determined. Additionally, the fruits were divided into marketable and unmarketable categories. Furthermore, measurements were made of both wet and dry weights.

### 2.8 Statistical analysis

The effects of *L. macroides* treatments on PRSV inhibition were examined using analysis of variance (ANOVA). All results were confirmed by doing the experiment three times. The web-based statistical application XLSTAT Pro statistical analysis software (Addinsoft, New York, NY, USA) was used to perform an analysis of variance on the experimental data. DMRT was used to compare treatment means.

## 3 Results

### 3.1 Identification of rhizobacterial isolate

**Figure 1:**
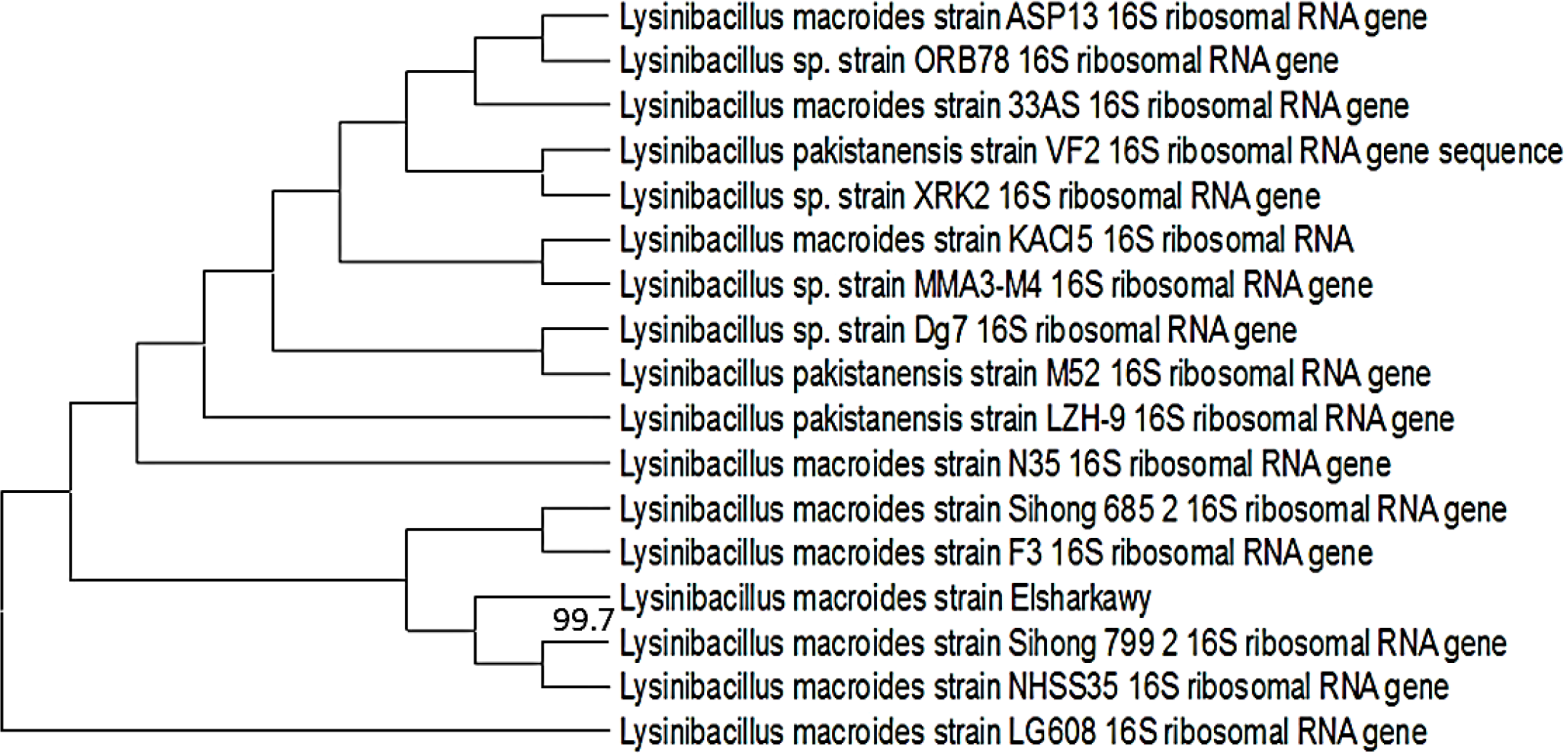
The Bootstrap neighbor-joining tree displays the phylogenetic relationship between the locally isolated *L. macroides* strain Elsharkawy and other isolates.

The antiviral effectiveness of around forty colonies with a variety of morphological characteristics was assessed (Data not shown). All of the bacterial isolates were used to develop biopriming treatments. The PRSV inoculation and disease severity evaluation were carried out as previously mentioned. For molecular characterisation, the bacterial isolate with the highest level of antiviral effectiveness was selected. The 16S rRNA sequence of the isolate was aligned with the NCBI database. A significant association between the strains was shown by the alignment, which confirmed a 99.7% identity with *L. macroides* (**Figure 1**). The findings of the sequence homology analysis indicated that the bacterial isolate was *L. macroides* strain Elsharkawy. Following its submission to GenBank, the annotated sequence acquired the accession number PX735745.

### 3.2 Effect of *L. macroides* treatments on PRSV severity

**Figure 2:**
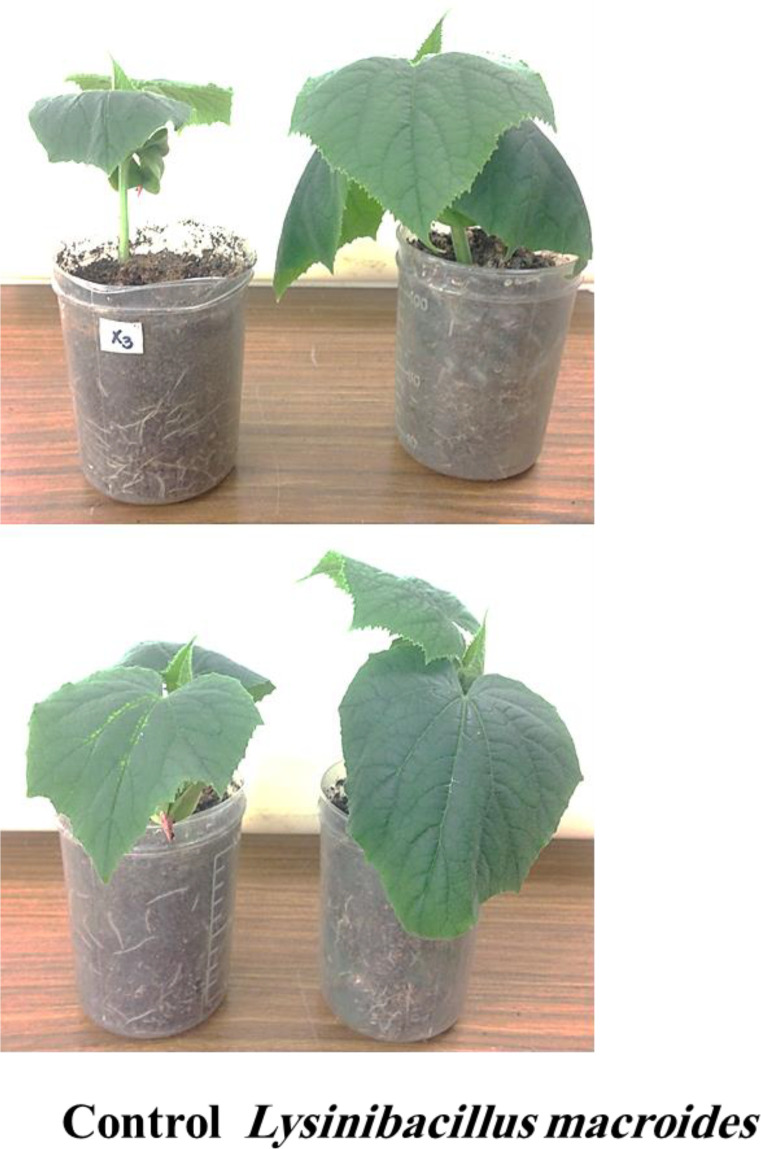
The impact of seed biopriming with *Lysinibacillus macroides* on the growth of cucumber plants at 2 and 3 weeks after planting.

**Figure 3:**
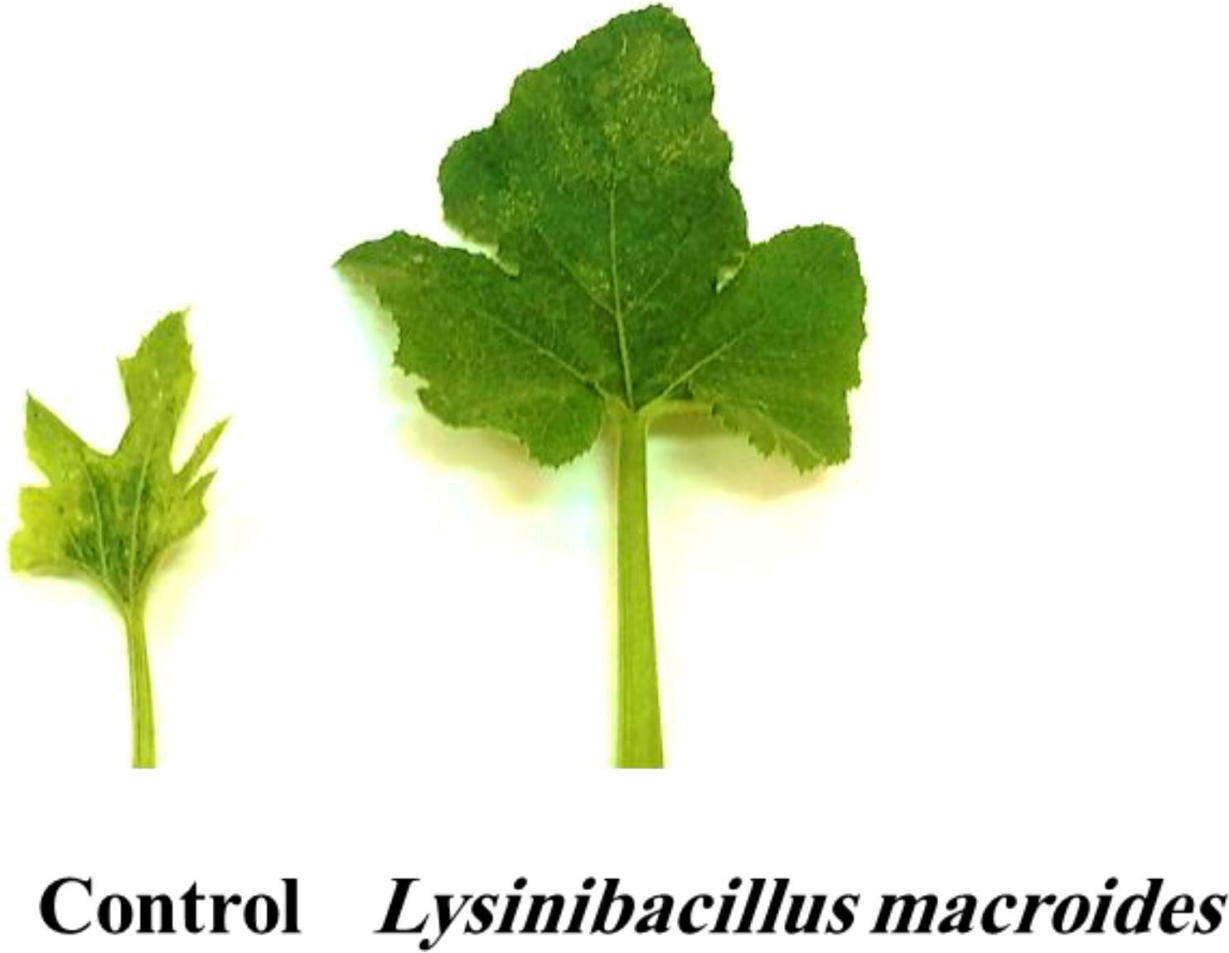
Characteristics of cucumber leaves following PRSV challenge in *Lysinibacillus macroides* SD treatment compared to infected control (exhibiting severe mosaic and deformed leaves) at three weeks post PRSV inoculation.

**Figure 4:**
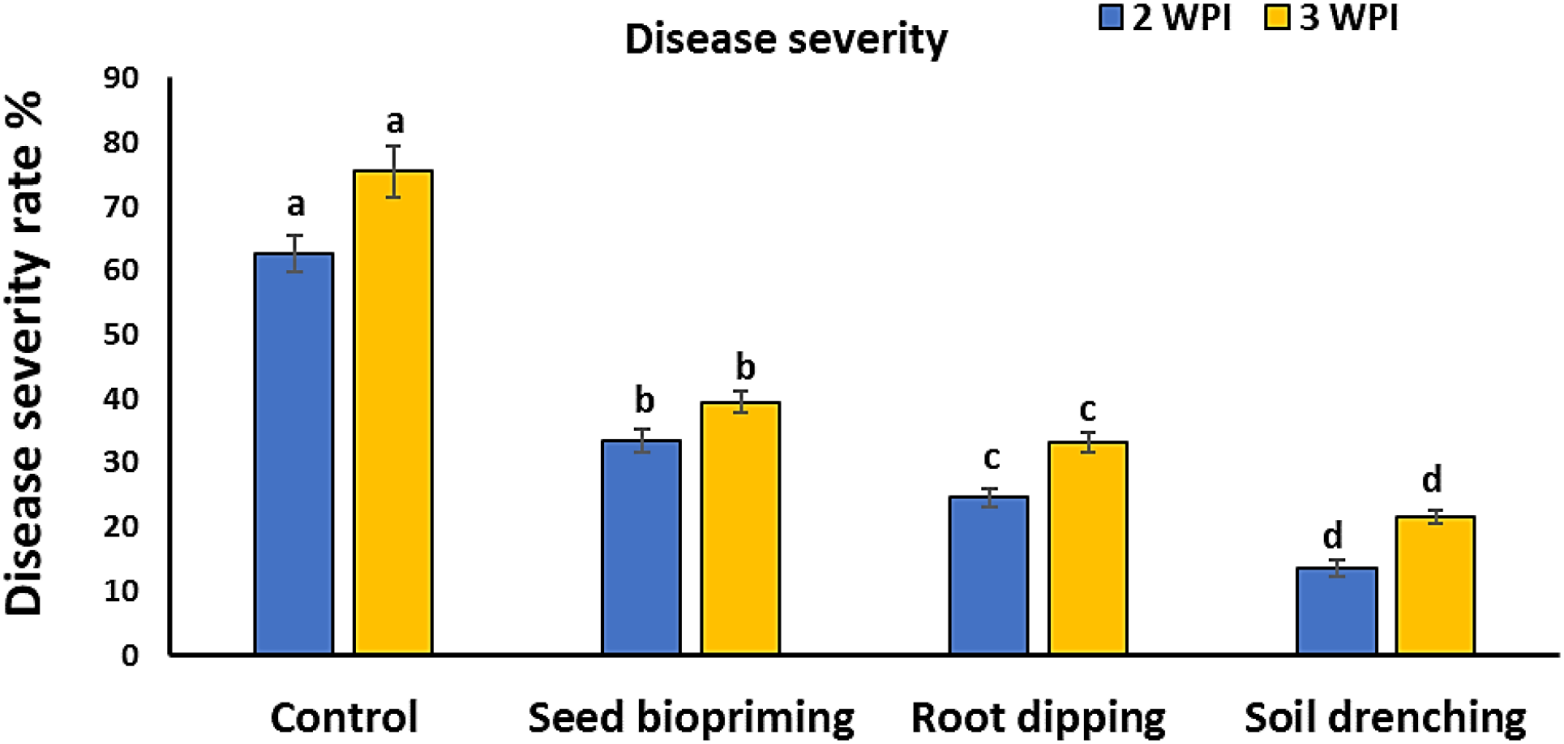
Impact of various application methods of *Lysinibacillus macroides* on the severity of papaya ringspot virus at 2- and 3- weeks post-inoculation. Different letters denote statistically significant differences among the treatments.

The application of *L. macroides* to healthy plants resulted in a markedly higher growth rate compared to the control group (**Figure 2**). *L. macroides*-treated soil significantly decreased PRSV symptoms in infected plants, but untreated control plants showed a substantial mosaic with tiny deformed leaves (**Figure 3**). PRSV severity was assessed at two and three WPIs, and all *L. macroides* administration techniques substantially decreased the disease severity (**Figure 4**). SD treatment with *L. macroides* was shown to be the most successful approach for lowering the severity of PRSV at 2 WPI. Additional application processes, including RD and SP, revealed considerable outcomes. The greatest levels of PRSV control at 3 WPI were attained with SD application (**Figure 4**). SD treatment showed significant results at 2- and 3-WPI, with values of 13.6% and 21.5%, respectively (**Figure 4**). However, RD showed 24.6% and 33.1% at 2- and 3-WPI, respectively. The SP treatment produced effectiveness rates of 33.4% and 39.4% at 2- and 3-WPI, respectively.

### 3.3 Effect of *L. macroides* application methods on PRSV accumulation

**Figure 5:**
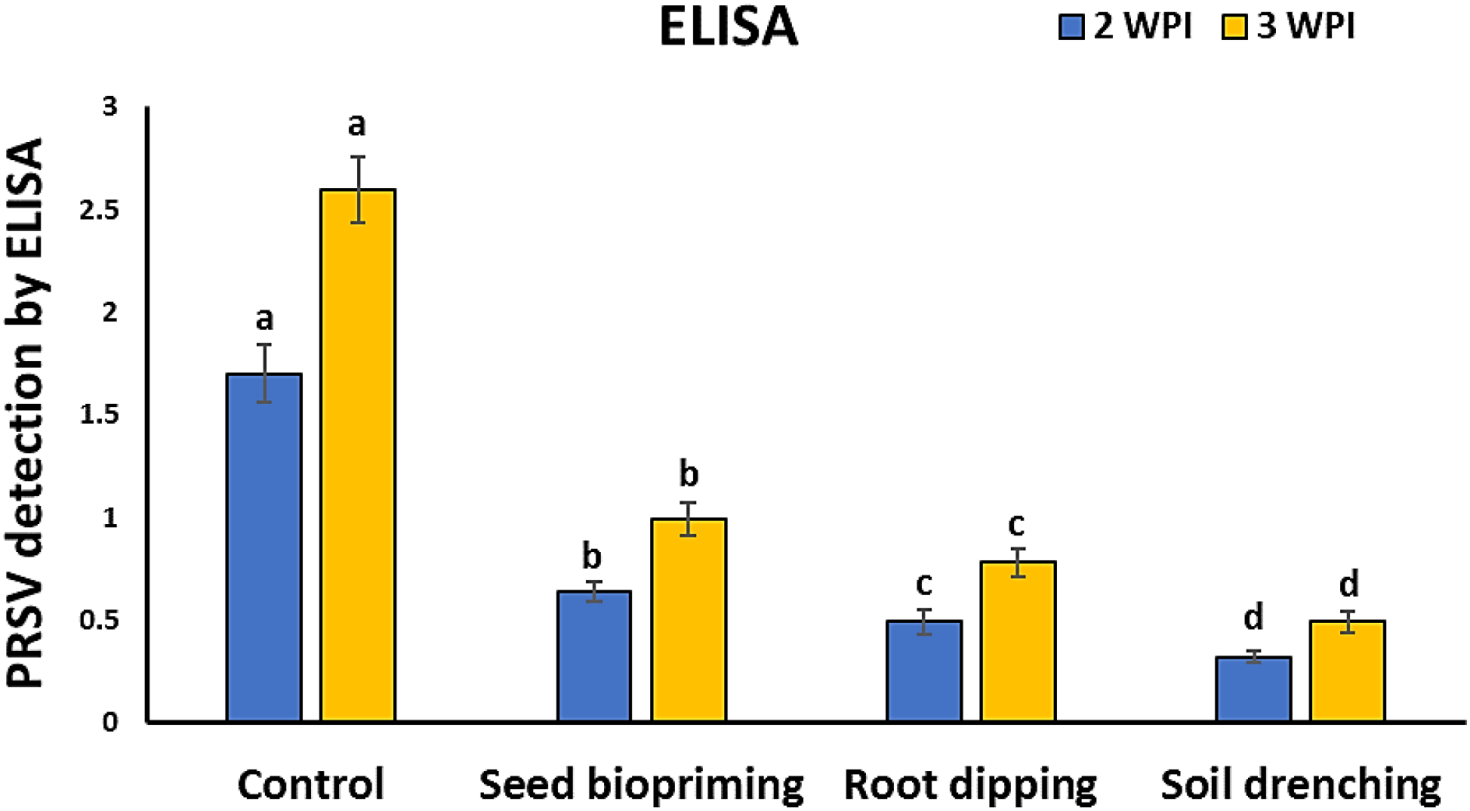
Impact of various application methods of *Lysinibacillus macroides* on PRSV concentration using ELISA at 2- and 3- weeks post-inoculation. Different letters denote statistically significant differences among the treatments.

In comparison to the control plants, all cucumber plants treated with *L. macroides* showed significantly less PRSV accumulation in their leaves as measured by ELISA at 2- and 3-WPI (**Figure 5**). *L. macroides*-SD-treated cucumber leaves showed significantly reduced levels of PRSV accumulation at 2 and 3 WPI compared to the other groups. A control treatment with an optical density (OD) value of 2.6 at 3 WPI measured by ELISA had the highest PRSV accumulation level, followed by *L. macroides*-SP with an OD value of 0.99, *L. macroides*-RD with an OD value of 0.78, and *L. macroides*-SD with an OD value of 0.49. (**Figure 5**).

### 3.4 Impact of *L. macroides* on total soluble carbohydrates and proteins

Protein levels increased to 1.8 mg/g DW and total soluble carbohydrates (TSC) decreased to 0.6 mg/g DW in the control group, compared to the healthy plants. Maximum TSC and protein content values of 2.0 mg/g DW and 2.4 mg/g DW, respectively, were achieved by *L. macroides*-SD application (**Table 2**).

**Table 2:** Assessment of total soluble carbohydrates and proteins in cucumber plants following PRSV infection, as influenced by *Lysinibacillus macroides* treatment.

Treatment	Total Soluble Carbohydrates mg/g DW*	Total Soluble Proteins mg/g DW
Healthy	10.9 ± 0.2 ^a^	2.5 ± 0.2 ^a^
*Lysinibacillus macroides*-SP	1.6 ± 0.1 ^c^	2.3 ± 0.2 ^a^
*Lysinibacillus macroides*-RD	1.7± 0.1 ^c^	2.4 ± 0.1 ^a^
*Lysinibacillus macroides*-SD	2.0 ± 0.1 ^b^	2.4 ± 0.2 ^a^
Infected	0.6 ± 0.1 ^d^	1.8 ± 0.1 ^b^

*Different letters denote statistically significant differences among treatments.

### 3.5 Effect of *L. macroides* on Ascorbic acid and free radicals scavenging

The capacity of *L. macroides* to stimulate the formation of antioxidant phytochemicals was evaluated by comparing the AsA level in the *L. macrolides*-treated and PRSV-challenged groups to the controls (**Table 3**). AsA levels in the infected cucumber (370 mg/g FM) were lower than those in the healthy group (820 mg/g FM). The AsA content was enhanced in all *L. macroides* treatments, but the *L. macroides* -SD group outperformed the infected cucumber by a significant margin, reaching 688 mg/g FM.

**Table 3:** Assessment of free radical scavenging and ascorbic acid production in cucumber plants following PRSV infection, as influenced by *Lysinibacillus macroides* treatment.

Treatment	DPPH (%) *	Ascorbic acid (mg/g FM)
Healthy	37 ± 1.7^d^	820 ± 13.8^a^
*Lysinibacillus macroides*-SP	64 ± 2.8^b^	564 ± 10.9^c^
*Lysinibacillus macroides*-RD	65 ± 2.4^b^	575 ± 11.5^c^
*Lysinibacillus macroides*-SD	77 ± 2.6^a^	688 ± 12.1^b^
Infected	58 ± 1.4^c^	370 ± 6.9^d^

*Different letters denote statistically significant differences among treatments.

FRS activity rose to 58% in the infected treatment group, which is greater than the healthy group’s 37% (**Table 3**). *L. macroides* treatment enhanced FRS in the *L. macroides*-SD (77%), *L. macroides*-RD (65%), and *L. macroides*-SP (64%) groups compared to infected plants. *L. macroides*-SD plants achieved the best performance in terms of FRS activity.

### 3.6 Assessment of superoxide dismutase, peroxidase and polyphenol oxidase activities

Infected plants showed a lower SOD level (0.11 µM/g F.W.) than the healthy treatment (**Table 4**). *L. macroides* increased SOD levels in all treatments. The *L. macroides*-SD group had the maximum SOD production at 0.18 µM/g F.W.

**Table 4:** Assessment of antioxidant enzymes production in cucumber plants following PRSV infection, as influenced by *Lysinibacillus macroides* treatment.

Treatment	Superoxide dismutase µM/g F. W. *	Polyphenol oxidase µM/g F. W.	Peroxidase µM/g F. W.
Healthy	0.16 ± 0.04^a^	0.32 ± 0.03^d^	0.43 ± 0.04^c^
*Lysinibacillus macroides*-SP	0.17 ± 0.03^a^	0.56 ± 0.04^c^	0.77 ± 0.04^b^
*Lysinibacillus macroides*-RD	0.17 ± 0.04^a^	0.68 ± 0.05^b^	0.79 ± 0.05^b^
*Lysinibacillus macroides*-SD	0.18 ± 0.03^a^	0.81 ± 0.06^a^	0.94 ± 0.06^a^
Infected	0.11 ± 0.02^b^	0.22 ± 0.03^e^	0.29 ± 0.02^d^

*Different letters denote statistically significant differences among treatments.

The PPO level was a little lower in the infected plants (0.22 µM/g F.W.) than in the healthy plants (0.32 µM/g F.W.) (**Table 4**). It might have originated from the cucumber’s first response to the PRSV infection. PPO levels were higher in the *L. macroides*-SD (0.81 µM/g F.W.) and *L. macroides*-RD (0.68 µM/g F.W.) groups, respectively, than in the infected group, indicating that *L. macroides* may promote PPO synthesis in cucumber plants after PRSV infection. The POX level of the infected plants 0.29 µM/g F.W was different from that of the healthy plants, which showed a level of 0.43 µM/g F.W. POX levels were significantly elevated in both the *L. macroides*-SD group (0.94 µM/g F.W.) and the *L. macroides*-RD group (0.79 µM/g F.W.) (**Table 4**).

### 3.7 Impact of *L. macroides* on relative expression of defense genes

**Figure 6:**
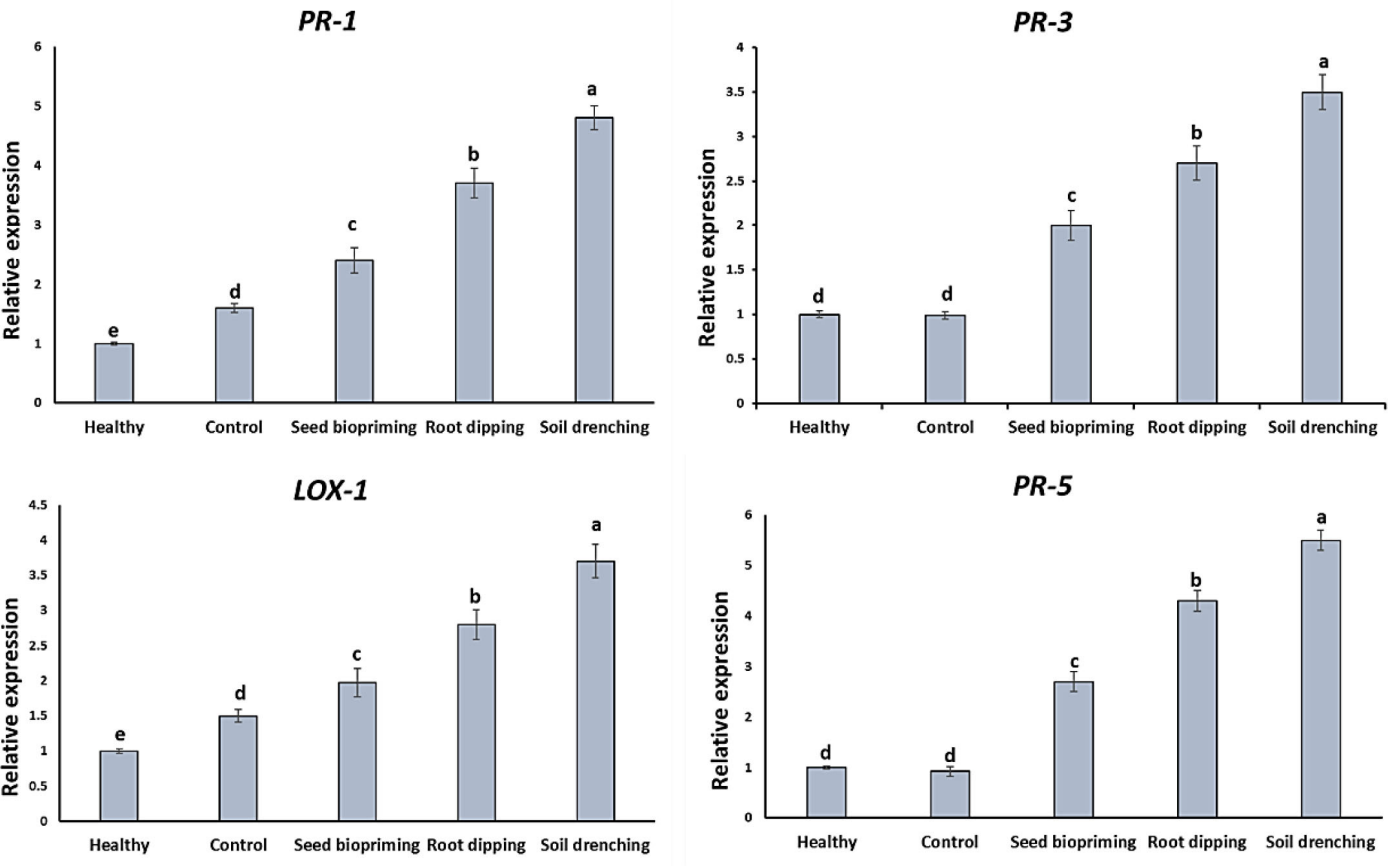
Relative expression of *PR-1, PR-3, PR-5* or *LOX-1* genes in cucumber treated with *Lysinibacillus macroides* after PRSV inoculation. Different letters denote statistically significant differences among treatments.

After PRSV infection in cucumber, the expression levels of PR genes were tracked to evaluate the effects of *L. macroides* treatments. The infected control group’s *LOX-1* expression was somewhat higher (1.4 folds) than the healthy group (**Figure 6**). This could be due to the fact that the cucumber plant’s first reaction was to launch the defense against the PRSV infection. The *LOX-1* expression levels in the *L. macroides*-SD, *L. macroides*-RD, and *L. macroides*-SP groups rose by 3.7, 2.8, and 1.97 times, respectively, after treatment with *L. macroides* (**Figure 6**). *L. macroides* stimulates the JA resistance pathway, as the peak *LOX-1* expression level was 3.7 times higher than that of the control group.

*PR-1, PR-3*, and *PR-5* levels were measured in each treatment group due to their importance in viral infection. *PR-1* expression was 1.5 times higher in the infected control group than in the healthy plants. This might be as a result of the cucumber plants’ first response to the PRSV infection, which produced a 1.5-fold increase in *PR-1* gene expression in the infected treatment group relative to the healthy group, as illustrated in **Figure (6)**. Each of the groups treated with *L. macroides* showed an increase in the expression of the *PR-1* and *PR-5* genes compared to the control group; the *L. macroides*-SD group had a 4.8- and 5.5-fold rise, respectively, while the *L. macroides*-RD group showed a 3.7- and 4.3-fold rise, respectively. Additionally, *PR-3* expression was slightly lower in the infected plants compared to the healthy plants. The expression of the *PR-3* gene was upregulated 3.5-, 2.7-, and 2-folds, following treatment with *L. macroides*-SD, *L. macroides*-RD, and *L. macroides*-SP, respectively, relative to the control (**Figure 6**).

### 3.8 Effect of *L. macroides* on growth and yield of cucumber plants

Infected plants demonstrated a notable reduction in the growth parameters relative to control plants. Infected plants treated with *L. macroides* exhibited a significant increase in leaf area and total green coloring relative to control plants (**Table 5**). **Table 5** indicates that infected leaves had significantly lower total green color (TGC) concentrations (23.9 and 26.2 in the first and second season, respectively) than the control. **Table 5** demonstrates that the infected cucumber plants treated with *L. macroides* raised TGC to 29.9 in the first season and 32.3 in the second season when compared to the untreated control plants. Likewise, leaf area was enhanced in *L. macroides* treated plants, measuring 57.5 and 67.9 dm² in the first and second seasons, respectively, in comparison to the control (**Table 5**). **Table 6** demonstrates that the total yield improved to 3.5 and 4.9 kg/plant in the first and second seasons, respectively, when compared to the infected control plants. *L. macroides*-treated cucumber plants produced only 1.5 and 1.7 kg of nonmarketable fruits per plant in the first and second seasons, respectively (**Table 6**). However, compared to the control plants, virus infection increased the percentage of unmarketable fruits to 1.8 and 2.1 kg/plant in the first and second seasons, respectively (**Table 6**).

**Table 5:** Effect of *Lysinibacillus macroides* treatment on growth parameters of cucumber plants under PRSV challenge inoculation.

Treatments	1^st^ season		2^nd^ season	
	Leaf area/plant (dm^2^) *	Total green color (SPAD)	Leaf area/plant (dm^2^)	Total green color (SPAD)
Healthy	84.6 ± 3.9^a^	35.9 ± 1.4^a^	97.4 ± 4.1^a^	38.4 ± 1.6^a^
*Lysinibacillus macroides*-SP	57.5 ± 1.8^b^	29.9 ± 1.2^b^	67.9 ± 2.8^b^	32.3 ± 1.4^b^
Infected	22.3 ± 1.1^c^	23.9 ± 1.1^c^	25.8 ± 1.3^c^	26.2 ± 1.0^c^

*Different letters denote statistically significant differences among treatments.

**Table 6:** Effect of *Lysinibacillus macroides* treatment on total yield parameters of cucumber plants under PRSV challenge inoculation.

Treatments	1^st^ season			2^nd^ season		
	Weight/plant (kg) *	Marketable fruits	Non-marketable fruits	Weight/plant (kg)	Marketable fruits	Non-marketable fruits
Healthy	3.9 ± 0.7^a^	2.9 ± 0.3^a^	1.0 ± 0.1^c^	5.1 ± 0.5^a^	4.2 ± 0.4^a^	0.9 ± 0.1^c^
*Lysinibacillus macroides*-SP	3.5 ± 0.5^a^	2.0 ± 0.2^b^	1.5 ± 0.1^b^	4.9 ± 0.4^a^	3.2 ± 0.3^b^	1.7 ± 0.1^b^
Infected	1.8 ± 0.2^b^	0.0 ± 0.0^c^	1.8 ± 0.1^a^	2.1 ± 0.2^b^	0.0 ± 0.0^c^	2.1 ± 0.1^a^

*Different letters denote statistically significant differences among treatments.

## 4 Discussion

Plant growth and disease resistance are impacted both directly and indirectly by regular microbes in the rhizosphere. Samples gathered from cucumber rhizosphere were investigated to find possible biocontrol agents that may inhibit PRSV infection. Based on the findings of the NCBI-BLAST analysis, the nucleotide sequence of the 16S rRNA for the isolate was revealed to be 99.7% identical to the sequences of other *L. macroides* isolates.

Rapid management of the viral infection is essential. Induced defensive responses effectively combated many plant-infecting viruses, including PRSV (Madhusudhan et al., 2011; Ashwini, 2015; Elsharkawy and Mousa, 2015). The effectiveness of microbial inoculants, including rhizobacteria, may stimulate plant growth and reduce the severity of infections caused by certain potyviruses (Ruwanthi et al., 2014). Elsharkawy and Mousa (2015) verified the effect of beneficial fungi in reducing the severity of viral infections in cucumber. Therefore, the purpose of this study was to investigate how novel microbial inoculants may reduce disease symptoms. *L. macroides* has never been used in the management of viral infections.

Stimulating systemic resistance might be a different strategy for preventing or reducing the impact of viral diseases. This study demonstrated that *L. macroides* treatments may reduce PRSV symptoms in cucumbers. The highest reduction in PRSV severity and concentration was achieved by drenching the soil with *L. macroides*, which was followed by root dipping and seed priming. The activity of beneficial microbes in plants, known as PGPR-mediated ISR, may be a contributing factor for the reduction of PRSV severity and concentration (Elsharkawy and Mousa, 2015; Abdalla et al., 2017; Elsharkawy et al., 2022).

Applying *L. macroides*-SD resulted in the highest TSC and protein contents. Reduced leaf size, one of the symptoms of a PRSV infection, may be caused by the reduction in photosynthesis and, thus, the overall amount of carbohydrates. Although all treatment groups had improvements in AsA levels after applying *L. macroides*, the *L. macroides*-SD group proved to be the most effective. Accumulation of AsA inhibits RNA virus replication and alleviates viral symptoms, suggesting that it does more than only eliminate free radical species (Wang et al., 2011; Fujiwara et al., 2016). Ascorbic acid is an effective non-enzymatic antioxidant that is essential for plant growth and defense (Gallie, 2013; Sofy et al., 2020). Although the exact mechanism by which AsA enhances plants’ ability to withstand infection remains unknown, the correlation between various viral infections and elevated levels of AsA and oxidative stress highlights its importance (Fujiwara et al., 2013). Employing the DPPH assay, we evaluated the antioxidant potential of cucumber plants after PRSV infection. Treatments with *L. macroides* exhibited higher FRS compared to the infected plants. Enhancing the capacity for free radical elimination constitutes a key aspect of the plant’s defense mechanism against microorganisms, with the purpose of mitigating the adverse effects of rapid increases in oxidative stress (Dumanović et al., 2021; Zhu et al., 2021).

Viral infections in plants frequently induce substantial oxidative stress. As a measure of defense, infected plants synthesize elevated levels of antioxidant enzymes to mitigate the presence of free radicals (Sofy et al., 2020; Dumanović et al., 2021). In the current study, three antioxidant enzymes were assessed in cucumber plants exposed to PRSV as indicators of oxidative stress. The primary mechanism for eliminating superoxide species (O^2−^) involves superoxide dismutase (SOD) generating hydrogen peroxide (H_2_O_2_), which is subsequently decomposed by plant glutathione peroxidase or catalases. The application of *L. macroides* resulted in increased SOD production across all treatments, which matches the findings of El-Gendi et al. (2022). This study demonstrates that *L. macroides* may continue to promote PPO synthesis in cucumber plants after a PRSV infection by demonstrating that PPO levels were greater in the *L. macroides*-SD, *L. macroides*-RD, and *L. macroides*-SP groups. In addition to its role as an oxygen buffer during photosynthesis, PPO has been shown to have protective properties in plant tissues (Bakhtawar et al., 2022). Lignin is a key component in the defensive mechanism against infections; it is synthesized when phenolic compounds use PPO to remove reactive oxygen species (Mohammadi and Kazemi, 2002). The POX levels were assessed in both the *L. macroides* treatments and control groups. Plant oxidase enzymes participate in a broad range of cellular physiological processes and defense mechanisms. The synthesis of lignin, the enhancement of cell wall cross-linking, and the suppression of pathogen entry and proliferation through reactive oxygen metabolism are all notably influenced by class III POX during plant infection conditions (Fan et al., 2022). A greater POX synthesis in the protected group compared to the control group explained the role of *L. macroides* in defense response. The results of the three enzyme experiments demonstrate that *L. macroides* may significantly mitigate the oxidative stress generated by PRSV infection, especially when administered before infection and acting primarily via the enzymatic pathway.

Plants control their own growth and defense mechanisms in reaction to biotic and abiotic stresses by overexpressing a number of PR genes (Elsharkawy et al., 2022). After being inoculated with PRSV, cucumber plants may primarily defend themselves via the SA pathway. The expressions of *PR-1* and *PR-5* were shown to be upregulated in the group that received *L. macroides* as a pre-infection prophylactic treatment, in contrast to the infected group. The *PR-1* is one systemic resistance measures that regulates plant infection defenses. This study’s results support earlier studies that revealed slight activation of the *PR-1* gene after infection with a virus (Elsharkawy et al., 2022). The *PR-1* gene plays an essential role in plant defense against different stresses, in addition to controlling development and growth in stress-free situations. In comparison to the control group, *L. macroides* treatments showed an increase in *PR-3* transcripts. Chitinase activity encoded by *PR-3* is overexpressed and linked to plant resistance mechanisms (Zhao and Li, 2021). There has been a lot of focus on *PR-3*’s role in fungus protection, but its activity to protect plants against viral infections is yet unknown (Mathew et al., 2021). The induction of PR genes during PRSV infection may be explained by this finding (Elsharkawy and Mousa, 2015). Multiple papers have shown increased chitinase activity in virus-infected plants, and our results are consistent with those observations (Abdelkhalek et al., 2022). In response to biotic stresses, *LOX-1* regulates several cellular defense processes in plants, including jasmonic synthesis (Elsharkawy et al., 2022). There was an increase in *LOX-1* transcripts in the *L. macroides* treatments compared to the control group.

Total green color (TGC) and leaf area were significantly reduced on infected leaves as compared to healthy plants or plants treated with *L. macroides*. Several studies have examined the plant and virus interactions. Palanisamy et al. (2009) found that total chlorophyll decreased by 64% on infected leaves. The use of *L. macroides* increased the yield of marketable fruits. The financial losses caused by viral diseases were also reduced as a result of GU 23–3 treatments, which significantly raised the number of marketable cucumbers and decreased the amount of unmarketable cucumbers (Khalaf et al., 2019).

The results showed that PGPR (*L. macroides*) is an effective method for controlling PRSV. The severity of PRSV symptoms was significantly controlled by *L. macroides*, leading to a reduction in cucumber production losses caused by the virus. As part of integrated management strategies, *L. macroides* might provide a new method for controlling PRSV.

## Data Availability

The raw data supporting the conclusions of this article will be made available by the authors, without undue reservation.
